# Occupational Exposures Associated with Life Expectancy without and with Disability

**DOI:** 10.3390/ijerph17176377

**Published:** 2020-09-01

**Authors:** Astrid de Wind, Ranu Sewdas, Emiel O. Hoogendijk, Allard J. van der Beek, Dorly J. H. Deeg, Cécile R. L. Boot

**Affiliations:** 1 Behavioural Science Institute, Radboud University, 6500 Nijmegen, The Netherlands; 2 Coronel Institute of Occupational Health, Amsterdam Public Health Research Institute, Amsterdam UMC, University of Amsterdam, 1081 Amsterdam, The Netherlands; 3 Department of Public and Occupational Health, Amsterdam Public Health Research Institute, Amsterdam UMC, VU University Amsterdam, 1081 Amsterdam, The Netherlands; r.sewdas@amsterdamumc.nl (R.S.); a.vanderbeek@amsterdamumc.nl (A.J.v.d.B.); crl.boot@amsterdamumc.nl (C.R.L.B.); 4 Department of Epidemiology and Biostatistics, Amsterdam Public Health Research Institute, Amsterdam UMC, VU University Amsterdam, 1081 Amsterdam, The Netherlands; e.hoogendijk@amsterdamumc.nl (E.O.H.); djh.deeg@amsterdamumc.nl (D.J.H.D.)

**Keywords:** healthy life expectancy, disability, occupational exposure, ageing, gender

## Abstract

Policies to extend working lives often do not take into account potentially important health inequalities arising from differences in occupational exposures. Little is known about which occupational exposures are associated with these inequalities. This study aims to examine differences in life expectancy without and with disability by occupational exposures. Longitudinal data (1992–2016) on disability and physical and psychosocial work demands and resources of 2513 (former) workers aged ≥55 years participating in the Longitudinal Aging Study Amsterdam were used. Gender specific life expectancies without and with disability by occupational exposures were calculated using multistate survival models. Women aged 55 years with high physical work demands had a lower life expectancy without disability than those with low exposure (1.02–1.57 years), whereas there was no difference for men. Men and women with high psychosocial work demands and resources had a longer life expectancy without disability than those with low exposure (1.19–2.14 years). Life expectancy with disability did not significantly differ across occupational exposures. Workers with higher psychosocial demands and resources and lower physical demands can expect to live more disability-free years. Information on occupational exposure helps to identify workers at risk for lower life expectancy, especially without disability, who may need specific support regarding their work environment.

## 1. Introduction

Life expectancy has increased in most Western countries. This increase, together with lower fertility rates and maturing of the baby boomers, has resulted in ageing of the population [1]. To counteract the financial effects of an ageing population on social security systems, many Western governments have implemented retirement reforms to stimulate older workers to prolong their working lives. This includes adjustments of the retirement age based on the increase in life expectancy. However, an increase in life expectancy does not always go together with an increase in healthy life expectancy (HLE) (e.g., [2]). In fact, a study by Deeg et al. [3] showed a decline in physically HLE between 1993 and 2016 in the Netherlands. Furthermore, the reforms aimed at raising the retirement age generally do not take into account potentially important socioeconomic health inequalities among workers in different occupations. 

Socioeconomic health inequalities are generally considered to arise from a combination of factors in the material, psychosocial, and behavioral domains [4]. The material domain includes, next to differences in financial resources, living conditions, and security of employment status, also differences in work characteristics. Unfavorable work characteristics cluster within individuals with low occupational levels, and they contribute to poor health (e.g., [5,6,7,8]) and lower life expectancy. As such, work characteristics can explain part of the social gradient in health and life expectancy. Occupational level is one of the indicators of socioeconomic position (SEP) that has often been linked to the social gradient in health [9]. To illustrate, Head et al. showed that people in higher occupational positions could expect to live more years in good health and without chronic diseases compared to those in lower occupational positions [10]. 

Although it is known that occupational level is related to health, relatively little is known about which specific occupational exposures produce socioeconomic inequalities in health and HLE. Platts et al. [11] showed that more physically demanding and dangerous work was associated with fewer life years spent in good self-rated health and without chronic disease among a sample of male gas and electricity workers in France. Magnusson Hanson et al. [12] showed that also poor psychosocial working conditions, i.e., job strain, was associated with fewer years spent in good self-rated health and free from chronic disease in Finland, France, Sweden, and the UK. To our knowledge, these are the only two studies investigating HLE in relation to occupational exposure. 

These studies have in common that partial HLEs were estimated, i.e., HLE within age range 50 to 75 years. Therefore, it is still unknown how occupational exposure might be related to total HLE, i.e., also beyond the age of 75 years. Furthermore, these studies both addressed life expectancy in good self-rated health and without chronic disease. Disability-free life expectancy (DFLE) is another common measure of HLE, combining information on life expectancy and disability [13]. This measure has not previously been linked to occupational exposure. Having a chronic disease does not necessarily mean that someone is disabled. However, experiencing disability might be more directly related to wellbeing, hospitalization, and death than the chronic disease itself [14]. 

The determination of life expectancy without and with disability by occupational exposure helps to gain insight into health inequalities in later life based on differences in occupational exposures during working life. We extend previous work in this field by (1) investigating total life expectancies instead of partial life expectancies, (2) operationalizing health with a measure of disability instead of self-rated health and chronic disease, and (3) relating it to a range of specific physical and psychosocial work demands and resources. The current study aims to explore differences in life expectancy without and with disability in (former) workers aged 55 years and older in association with exposure to a range of physical and psychosocial work demands and resources. Women live longer than men in most countries in the world and, on average, have work exposures different from men, also in the Netherlands [15]. Therefore, we estimated life expectancies for men and women separately. By doing so, the current study contributes to identification of workers at risk for a lower life expectancy without or with disability.

## 2. Materials and Methods 

### 2.1. Dataset and Study Sample

We used data from the Longitudinal Aging Study Amsterdam (LASA). This interdisciplinary cohort study in the Netherlands aims to determine predictors and consequences of changes in functioning with aging. In 1992, a nationally representative sample of adults aged 55–85 years was invited to take part in the study. The initial response rate was 60% (*n* = 3107). Since its start, trained interviewers conducted examinations and interviews, among others on work (e.g., occupation) and health (e.g., disability) in respondents’ homes every three years. The VU University Medical Center Medical Ethical Committee has approved the LASA study (IRB numbers: 92/138, 2002/141, 2012/361, and 2016.301). Informed consent was obtained from all participants. More details about LASA can be found elsewhere [16,17]. 

For the current study, we used all eight measurement cycles available so far, that is, data from 1992 up to 2016. Inclusion criteria were having a paid job at baseline or having had a paid job earlier in life, leaving 2590 respondents. Participants who had missing information on disability or vital status at follow-up were excluded (*n* = 77). This resulted in a study sample of 2513 persons, including 1363 men and 1150 women.

### 2.2. Measures

#### 2.2.1. Vital Status

Mortality status and date of death were retrieved from the municipal population register. This register includes, among others, data on name, address, and dates of birth and death of Dutch residents.

#### 2.2.2. Disability

Disability was assessed using six questions selected from the validated Organization for Economic Cooperation and Development Questionnaire [18]. The six questions concerned difficulty in climbing or descending stairs of 15 steps without stopping, getting dressed and undressed, sitting down and standing up from a chair, cutting one’s toenails, walking outside for five minutes, and using own or public transportation. Questions could be answered on a 5-point scale, ranging from “no difficulty” to “not able”. Respondents who had (some) difficulty with at least one activity were classified as having disability. 

#### 2.2.3. Occupational Exposures

Occupational exposures were assessed by applying the general population job exposure matrix (GPJEM) on the longest held occupation of the participant. This GPJEM was developed by Rijs et al. [19] by linking self-reported physical and psychosocial work exposures among 55–64-year-old (former) workers to the Netherlands Standard Classification of Occupations 1992 (NSCO92) using data from the Netherlands Working Conditions Survey [20]. Occupational classes were categorized into low or high level of probability of exposure to several physical and psychosocial work demands and resources. Physical work demands included repetitive movements, use of force, and work in uncomfortable position. Psychosocial work demands included cognitive demands, task requirements, and time pressure. Psychosocial work resources included variation in activities and autonomy. More detailed information on development of the GPJEM and underlying measurements of psychical and psychosocial work exposures can be found elsewhere [19].

#### 2.2.4. Demographics

Age at the time of the interview was based on interview date and birthdate, which was obtained from municipal registries, as was gender. Highest level of education completed comprised three levels: low (elementary school or less, lower vocational education), intermediate (general intermediate, intermediate vocational, general secondary education), and high (higher vocational education, college, university). Level of education was used to describe the study sample and for sensitivity analyses (see below). Due to statistical power issues level of education was not included in the main analyses.

### 2.3. Statistical Analyses

**Figure 1:**
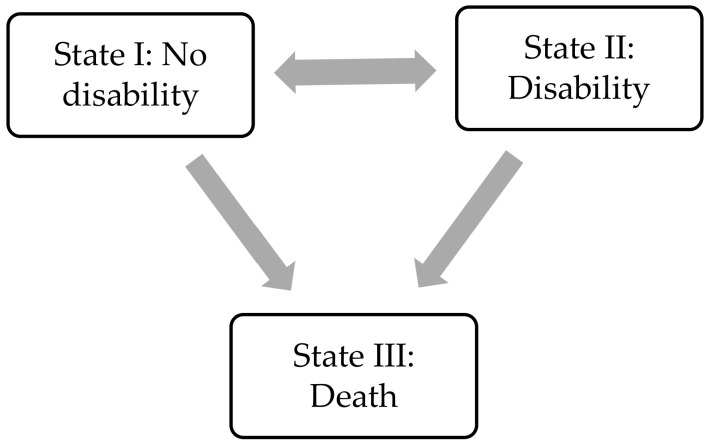
Three-state model. The figure illustrates the three states, i.e., (I) “No disability”, (II) “Disability”, and (III) “Death”, as well as the four possible transitions between the different states.

Baseline characteristics (i.e., means, SDs, frequencies and percentages) were examined. Subsequently, we estimated life expectancies without and with disability based on multi-state survival models. This method is based on incidence and recovery rates of disability and incidence of death. Three states were distinguished: (I) “No disability”, (II) “Disability”, and (III) “Death” (see Figure 1). In addition to the occupational exposures, age and gender were included as covariates in the models, where age was a time-varying covariate. The first step in the analyses was to model the transition probabilities between these states using a continuous-time three-state survival model with the R-Package Multistate Modelling (MSM). In this model the time of the transitions between states I and II were assumed to lie halfway between two measurement cycles. States I and II were living states, and thus, transitions may take place back and forth and several times. State III was an absorbing state, as this state could be entered only once. The exact transition time to this state was obtained from the data. Hazards were estimated for the effects of occupational exposures and covariates on transitions between the states. Hazard ratios (HR) and transition probabilities were derived from these hazards. 

In the second step, transition probabilities were used to estimate total life expectancies (LE) at the age of 55 years as well as LE without and with disability using the R-package Estimating Life Expectancies using Continuous Time (ELECT) [21]. LEs are reported separately for low and high exposure to any of the physical and psychosocial work demands and resources and separately for men and women. In line with previous studies, differences between LEs without and with disability were considered statistically significant if the point estimate of one LE was outside the 95% CI of the other LE and the other way around [22,23].

#### Sensitivity Analyses 

A common procedure in studies relating occupational exposure to health is to either adjust the analyses for (an indicator of) socioeconomic position (SEP) or to report results stratified by different levels of SEP (e.g., [12]). Statistical power did not allow stratification by gender and SEP at the same time. Therefore, in sensitivity analyses, we performed models that only included age and SEP (i.e., educational level) as covariates to be able to assess the LE differences by levels of SEP.

## 3. Results

### 3.1. Baseline Characteristics

Table 1 shows baseline characteristics for men and women. The average age was 70.6 for men and 69.6 for women. At baseline, 33.7% of the men and 44.4% of the women had disability. 

### 3.2. Total Life Expectancies and Life Expectancies without and with Disability

Total life expectancy for men aged 55 years was 21.16 years, of which 12.30 without disability and 8.86 with disability (Table 2). Total life expectancy for women aged 55 years was 26.36 years, of which 11.35 without disability and 15.00 with disability (Table 2). Thus, women had a higher total life expectancy than men but also spent more time with disability.

### 3.3. Physical Work Demands

Total life expectancy and life expectancy with disability of men at age 55 years did not significantly differ between high or low values of any physical work demand (Table 2). Nevertheless, those with a low need to use force at work could expect to live over one more year without disability, i.e., 13.11 years among men with low use of force compared to 12.04 years among those with high use of force. Among women at age 55 years, those with physically strenuous jobs regarding repetitive movements and use of force had a lower total life expectancy than those with less strenuous jobs regarding these aspects (26.11 years compared to 27.98 for repetitive movements, and 25.34 years compared to 27.15 for use of force). Need to work in uncomfortable position made no difference. Women with physically strenuous jobs regarding all physical work demands could expect to live fewer years without disability, i.e., with differences ranging from 1.02 to 1.57 years. Life expectancy with disability was not significantly different for high and low values of any physical work demand neither for men nor for women. Overall, differences in life expectancy without disability associated with physical work demands were larger for women than for men.

### 3.4. Psychosocial Work Demands

Men aged 55 years with high task requirements and time pressure had a longer total life expectancy than those with low values of these psychosocial work demands (Table 2). Differences were mainly due to years without disability. Among men with high task requirements this was 13.75 years compared to 11.83 among those with low task requirements. Among men with high time pressure, this was 13.95 years compared to 11.92 among those with low time pressure. Total life expectancy was not significantly different between men with high or low cognitive demands. Nevertheless, life expectancy without disability among men with high cognitive demands was 13.26 years compared to 11.99 among those with low cognitive demands. Among women, we found the same pattern, i.e., women with high values of all psychosocial work demands could expect to live more years in general (differences ranging from 1.56 to 1.99 years), which was mainly due to more years without disability (differences ranging from 1.19 to 1.93 years). Life expectancy with disability was not significantly different for high or low values of any psychosocial work demand both among men and women. Overall, differences in life expectancy without disability associated with psychosocial work demands were larger for women than for men.

### 3.5. Psychosocial Work Resources

Total life expectancy at age 55 years among men with high variation in activities and high autonomy was significantly higher than among men with low values of these psychosocial work resources (23.15 years compared to 20.73 for variation in activities and 22.13 years compared to 21.00 for autonomy, Table 2). These differences were mainly due to more years without disability, which was 14.01 years among men with high variation and 11.87 among those with low variation. Men aged 55 years with high autonomy could expect to live 13.40 years without disability compared to 11.66 for men with low autonomy. Among women, we found more or less the same pattern; women with high variation in activities had higher total life expectancy and life expectancy without disability than women with low variation in activities (28.15 years compared to 25.87 for total life expectancy, 13.11 years compared to 11.05 for life expectancy without disability). Total life expectancy was not significantly different for women with high or low autonomy, but life expectancy without disability was higher for women with high autonomy (12.61 years) than for women with low autonomy (10.79 years). Life expectancy with disability was not significantly different for high or low values of any psychosocial work resource both among men and women. Overall, differences in total life expectancy associated with psychosocial work resources were larger for men than for women.

### 3.6. Sensitivity Analyses

In the models stratified by educational level, the differences in life expectancy between occupational exposures were attenuated (Supplementary File—Table S1). That is, the sensitivity analyses showed that except for autonomy, high or low occupational exposure did not make a difference regarding total life expectancy, and life expectancy without and with disability. Life expectancy with disability at age 55 years among workers with low autonomy was greater than among those with high autonomy, regardless of educational level.

## 4. Discussion

This study explored differences in life expectancy without and with disability in (former) workers aged 55 years and older in association with exposure to a range of physical and psychosocial work demands and resources. In general, people in jobs with lower physical work demands, higher psychosocial work demands, and greater psychosocial work resources could expect to live more disability-free years. Total life expectancy differed for some of the work characteristics, and life expectancy with disability hardly differed by occupational exposure. 

The finding that women aged 55 years could expect to live longer than men and spend more time with disability has been reported before and has been referred to as the male-female health-survival paradox [24,25]. Our results suggest that part of these differences can be attributed to differences in occupational exposures. To illustrate, the difference in life expectancy between high and low occupational exposure was larger for women than for men. Moreover, especially differences in life expectancy without disability associated with physical and psychosocial work demands were larger for women than for men. On the other hand, differences with regard to psychosocial work resources were more pronounced among men than among women. 

Our finding that people, especially women, who were exposed to high physical work demands during their working life could expect to live fewer years without disability than those with low physical work demands is partially in line with previous work [11], which has shown that more physically demanding work was associated with fewer life years spent in good self-rated health and without chronic disease between the ages of 50 and 75 years among men in a French cohort. Our study, however, showed that differences in life expectancy without disability were more pronounced among women than among men. Based on this previous work [11], we would have expected larger differences among men as well. 

Our finding that the psychosocial work environment also relates to the number of years without disability is also in line with previous research on individuals from four cohort studies [12], which has shown that workers with high job strain could expect to live fewer years in good health. Upon a closer look, however, the results are more difficult to compare, as in this previous study job strain was defined as a combination of high job demands and low job control. To the knowledge of the authors, the current study is the first study to investigate separate, specific aspects of the psychosocial work environment in association with disability-free life expectancy, revealing that people in jobs with high cognitive demands, task requirements, and time pressure (i.e., psychosocial work demands), as well as high variation in activities and autonomy (i.e., psychosocial work resources) could expect to live more years without disability. Our study furthermore reveals that there are differences between men and women with regard to the association between the psychosocial work environment and life expectancy without disability. Although both men and women, with higher psychosocial work demands could expect to live more disability-free years, these differences in life expectancy are larger among women than among men. Gender differences, such as differences between masculine and feminine occupations and occupational exposures may play a role in these differences in disability-free life expectancy between men and women [26]. More research is needed to explore the role of occupational exposures in the male-female health-survival paradox.

When stratifying the analyses by educational level, exposure to most work characteristics did not show significant differences in total life expectancy and life expectancy without disability. This is not surprising as specific occupational exposures cluster within educational levels. People with a higher educational level, in general, reach a higher occupational level, which is typically associated with lower physical job demands and higher job control [27]. 

### 4.1. Public Health Implications

Although the current study does not provide insight into causality with regard to occupational exposures and (healthy) life expectancy, occupational markers of lower (healthy) life expectancy may, in themselves, provide valuable information on which groups should be targeted in policies to extend working lives to prevent occupational exposure based socioeconomic health inequalities. We identified workers who may need specific support regarding their work environment. Moreover, some workers may benefit from more flexible pension schemes. To illustrate, the possibility for earlier pension take up may prevent workers with adverse occupational exposures from the potentially negative health consequences of extended working lives. At the same time, the possibility for partial retirement may allow people to work longer by providing more time for recovery during a working week. 

Nevertheless, when developing policies or measures targeting specific vulnerable groups in the labor market, it is important to also take into consideration the larger context of socioeconomic health inequalities. Work is one domain explaining socioeconomic health inequalities that are, in general, considered to originate from a combination of factors in the material domain (including work), the psychosocial domain, and the behavioral domain [4]. Besides adverse working conditions, the material domain includes poor housing, insecure employment, and a poor financial situation. The psychosocial domain includes negative life events, chronic strain, and low mastery, coping and social support. The behavioral domain includes unhealthy behavior, such as substance use, lack of physical activity and poor dietary habits [28]. A systematic review by Dieker et al. [29] showed that physical and psychosocial work factors explained approximately one-third of the socioeconomic inequalities in self-rated health, irrespective of lifestyle, which explained approximately one-fifth of the socioeconomic inequalities in self-rated health. 

### 4.2. Methodological Considerations

A strength of our study is that we used prospective data with a follow-up of more than two decades. Another strength is that members of the LASA cohort constitute a representative sample of the older population in the Netherlands. Nevertheless, the total life expectancy for men and women in our study was somewhat lower than estimations by Statistics Netherlands for the same age group and period, i.e., 21.16 years compared to 22.55 for men, and 26.35 years compared to 27.76 for women [30]. Furthermore, it should be noted that health-based selection into occupations with certain work characteristics may have taken place. Healthy workers are more likely to remain employed in more demanding jobs up to older ages than those who are less healthy. This may have resulted in an underestimation of the differences in total life expectancy and life expectancy without disability based on occupational exposures. Related to this, misclassification regarding occupational exposure may have taken place. By applying a GPJEM, within-occupation differences as well as changes in occupation throughout the working life are not taken into account. Furthermore, a validation study showed that the GPJEM that we applied classifies jobs according to probability of exposure to physical work demands more accurately than to psychosocial work demands and resources [18]. Moreover, the GPJEM was developed based on work exposure in 2005–2010 on a population of workers in 1992. This may have resulted in an overestimation of the psychosocial work demands and an underestimation of the physical work demands for the earlier years that were included in our multistate models. Nevertheless, applying a JEM is considered a useful way of assigning occupational exposures to individuals in case of lacking information on the individual level [31].

## 5. Conclusions

In conclusion, we found differences in total life expectancy as well as life expectancy without disability by occupational exposure. People in jobs with higher psychosocial work demands and resources and lower physical work demands could expect to live more years without disability. Regarding psychosocial work demands and resources, male and female workers exposed to low task requirements, low time pressure and low variation in activities could expect to have a shorter total life expectancy as well as a shorter life expectancy without disability from age 55 onwards. Information on occupational exposure helps to identify workers at risk for a lower life expectancy, especially without disability, who may need specific support regarding their work environment or who may benefit from more flexible pension schemes.

## Figures and Tables

**Table 1:** Baseline characteristics by sex [SD = standard deviation].

	Men (*n* = 1363)	Women (*n* = 1150)
	Mean (SD)/*N* (%)	Mean (SD)/*N* (%)
Age	70.6 (8.7)	69.6 (8.8)
Educational level		
Low	761 (55.8%)	749 (65.3%)
Intermediate	392 (28.8%)	295 (25.7%)
High	210 (15.4%)	103 (9.0%)
Ethnicity		
Dutch	1349 (99.0%)	1140 (99.1%)
Other	14 (1.0%)	10 (0.9%)
Disability		
No	903 (66.3%)	639 (55.6%)
Yes	460 (33.7%)	511 (44.4%)
Physical work demands		
Repetitive movements		
Low	338 (24.8%)	197 (17.1%)
High	1025 (75.2%)	953 (82.9%)
Use force		
Low	554 (40.6%)	510 (44.3%)
High	809 (59.4%)	640 (55.7%)
Uncomfortable Position		
Low	556 (40.8%)	510 (44.3%)
High	807 (59.2%)	640 (55.7%)
Psychosocial Work Demands		
Cognitive demands		
Low	1035 (75.9%)	962 (83.7%)
High	328 (24.1%)	188 (16.3%)
Task Requirements		
Low	1062 (77.6%)	950 (82.6%)
High	301 (22.1%)	200 (17.4%)
Time pressure		
Low	1060 (77.8%)	950 (82.6%)
High	303 (22.2%)	200 (17.4%)
Psychosocial Work Resources		
Variation in Activities		
Low	1106 (81.1%)	965 (83.9%)
High	257 (18.9%)	185 (16.1%)
Autonomy		
Low	673 (49.4%)	945 (82.2%)
High	690 (50.6%)	205 (17.8%)

**Table 2:** Total life expectancy and life expectancy without and with disability in association with occupational exposure of the workers at the age of 55 years for men and women (95% CI = 95% confidence interval).

	Men (*n* = 1363)			Women (*n* = 1150)		
	Total Life Expectancy in Years (95% CI)	Life Expectancy without Disability in Years (95% CI)	Life Expectancy with Disability in Years (95% CI)	Total Life Expectancy in Years (95% CI)	Life Expectancy without Disability in Years (95% CI)	Life Expectancy with Disability in Years (95% CI)
Overall	21.16 (20.13, 22.12)	12.30 (11.65, 12.99)	8.86 (8.21, 9.46)	26.36 (25.34, 27.25)	11.35 (10.66, 11.96)	15.00 (14.09, 15.72)
Physical work demands						
Repetitive movements						
Low	22.37 (20.29, 23.85)	13.06 (11.80, 14.14)	9.31 (8.06, 10.36)	27.98 (26.12, 28.82)	12.18 (11.21, 12.89)	15.79 (14.28, 16.82)
High	20.75 (19.44, 21.73)	12.03 (11.26, 12.80)	8.72 (7.78, 9.36)	26.11 (24.68, 26.64)	11.16 (10.48, 11.57)	14.95 (13.84, 15.70)
Use force						
Low	21.89 (20.06, 22.89)	13.11 (12.09, 13.92)	8.77 (7.63, 9.40)	27.15 (25.63, 28.02)	12.05 (11.03, 12.87)	15.09 (13.88, 16.20)
High	21.10 (19.97, 22.08)	12.04 (11.28, 12.78)	9.06 (8.24, 9.72)	25.34 (24.30, 26.04)	10.48 (9.71, 11.15)	14.86 (13.87, 15.61)
Uncomfortable position						
Low	21.68 (20.37, 22.91)	13.16 (12.23, 13.93)	8.52 (7.68, 9.40)	26.62 (25.30, 27.63)	11.79 (11.01, 12.44)	14.83 (13.46, 15.70)
High	21.54 (20.31, 22.34)	12.43 (11.66, 13.09)	9.11 (8.35, 9.81)	25.45 (24.42, 26.30)	10.48 (9.71, 11.11)	14.97 (14.10, 15.83)
Psychosocial work demands						
Cognitive demands						
Low	20.79 (19.45, 21.79)	11.99 (11.09, 12.82)	8.80 (7.95, 9.39)	26.01 (25.09, 26.88)	11.07 (10.51, 11.87)	14.94 (14.22, 15.54)
High	22.38 (20.38, 23.66)	13.26 (12.18, 13.97)	9.12 (7.80, 10.11)	27.57 (26.06, 28.68)	12.26 (11.23, 13.39)	15.31 (14.08, 16.43)
Task requirements						
Low	20.36 (19.02, 21.44)	11.83 (11.13, 12.41)	8.53 (7.70, 9.30)	25.54 (24.47, 26.32)	11.33 (10.69, 11.93)	14.21 (13.25, 15.12)
High	22.97 (21.07, 24.20)	13.75 (12.49, 14.68)	9.22 (8.27, 10.27)	27.46 (26.10, 28.29)	12.82 (11.88, 13.65)	14.64 (13.46, 15.59)
Time pressure						
Low	21.00 (19.70, 21.94)	11.92 (11.08, 12.68)	9.08 (8.38, 9.71)	25.56 (24.42, 26.25)	10.85 (10.27, 11.49)	14.71 (13.67, 15.39)
High	23.18 (21.28, 24.24)	13.95 (12.91, 14.80)	9.23 (8.09, 10.09)	27.55 (26.07, 28.63)	12.78 (11.86, 13.73)	14.77 (13.48, 15.72)
Psychosocial work resources						
Variation in activities						
Low	20.73 (19.39, 21.49)	11.87 (11.07, 12.44)	8.87 (8.18, 9.52)	25.87 (24.36, 26.69)	11.05 (10.29, 11.53)	14.82 (13.83, 15.74)
High	23.15 (21.01, 24.95)	14.01 (12.83, 15.11)	9.14 (7.72, 10.33)	28.15 (26.41, 29.25)	13.11 (12.11, 14.54)	15.05 (13.44, 16.14)
Autonomy						
Low	21.00 (19.71, 22.10)	11.66 (10.85, 12.33)	9.34 (8.42, 10.32)	26.14 (25.43, 26.88)	10.79 (10.36, 11.37)	15.35 (14.51, 15.94)
High	22.13 (21.05, 23.18)	13.40 (12.65, 14.28)	8.73 (7.94, 9.38)	27.20 (25.98, 28.17)	12.61 (11.68, 13.54)	14.59 (13.62, 15.66)

## Supplementary Materials

The following are available online at https://www.mdpi.com/1660-4601/17/17/6377/s1. Table S1: Total life expectancy and life expectancy without and with disability in association with occupational exposure of the workers at the age of 55 years for low, intermediate, and high educational levels.
