# Supplementary material for: Occupational Exposures Associated with Life Expectancy without and with Disability
Source: Int J Environ Res Public Health. 2020 Sep 1;17(17):6377. doi: 10.3390/ijerph17176377 (PMC7503628; doi:10.3390/ijerph17176377)
Supplement: Supplementary file 1 [file ijerph-17-06377-s001.zip › ijerph-887538-supplementary/ijerph-887538-supplementary.docx]

Supplementary Material:

**Table S1.** Total life expectancy, and life expectancy without and with disability in association with occupational exposure of the workers at the age of 55 years for low, intermediate and high educational level [95% CI=95% confidence interval].

|  | **Low educational level (n=761)** | | | **Intermediate educational level (n=392)** | | | **High educational level (n=210)** | |  |
| --- | --- | --- | --- | --- | --- | --- | --- | --- | --- |
|  | **Total life expectancy in years (95% CI)** | **Life expectancy without disability in years**  **(95% CI)** | **Life expectancy with disability in years**  **(95% CI)** | **Total life expectancy in years**  **(95% CI)** | **Life expectancy without disability in years**  **(95% CI)** | **Life expectancy with disability in years**  **(95% CI)** | **Total life expectancy in years**  **(95% CI)** | **Life expectancy without disability in years**  **(95% CI)** | **Life expectancy with disability in years**  **(95% CI)** |
| Overall | 22.40  (21.18, 23.31) | 10.27  (9.66, 10.93) | 12.13  (11.01, 12.86) | 23.43  (22.69, 23.96) | 11.79  (11.22, 12.22) | 11.64  (11.11, 12.19) | 24.54  (23.42, 25.32) | 13.40  (12.75, 13.98) | 11.14  (10.18, 11.81) |
| **Physical work demands** | | | | | | |  |  |  |
| *Repetitive movements* | |  |  |  |  |  |  |  |  |
| Low | 22.85  (20.29, 24.66) | 10.49  (9.55, 11.70) | 12.37  (10.51, 13.75) | 23.79  (21,79, 24.98) | 11.96  (10.94, 12.82) | 11.83  (10.42, 12.81) | 24.81  (23.11, 26.18) | 13.51  (12.54, 14.43) | 11.29  (10.15, 12.31) |
| High | 22.45  (21.28, 23.32) | 10.31  (9.55, 11.09) | 12.13  (11.07, 12.88) | 23.36  (22.52, 23.95) | 11.76  (11.07, 12.29) | 11.59  (10.97, 12.17) | 24.34  (23.27, 25.26) | 13.30  (12.45, 14.05) | 11.04  (10.04, 12.04) |
| *Use force* |  |  |  |  |  |  |  |  |  |
| Low | 23.08  (20.57, 24.36) | 10.77  (9.81, 11.43) | 12.31  (10.24, 13.82) | 23.72  (22.48, 24.45) | 12.08  (11.53, 12.70) | 11.63  (10.73, 12.29) | 24.37  (22.96, 25.40) | 13.42  (12.55, 14.11) | 10.94  (9.84, 11.82) |
| High | 22.76  (21.03, 23.25) | 10.31  (9.37, 10.86) | 12.45  (11.14, 13.07) | 23.56  (21.98, 24.16) | 11.68  (10.91, 12.25) | 11.88  (10.65, 12.45) | 24.38  (22.63, 25.51) | 13.09  (12.10, 13.80) | 11.29  (9.79, 12.21) |
|  |  |  |  |  |  |  |  |  |  |
| *Uncomfortable position* | |  |  |  |  |  |  |  |  |
| Low | 23.21  (20.93, 24.51) | 10.64  (9.48, 11.54) | 12.57  (11.03, 13.42) | 23.84  (22.86, 24.74) | 12.11  (11.46, 12.71) | 11.74  (11.00, 12.68) | 24.46  (23.27, 25.38) | 13.59  (12.66, 14.29) | 10.87  (10.05, 11.62) |
| High | 22.57  (20.33, 23.31) | 10.20  (9.37, 10.84) | 12.36  (10.81, 13.13) | 23.29  (22.14, 23.97) | 11.68  (11.05, 12.39) | 11.61  (10.64, 12.13) | 24.03  (22.39, 24.94) | 13.20  (12.03, 14.24) | 10.82  (9.48, 11.52) |
| **Psychosocial work demands** | | | | | | |  |  |  |
| *Cognitive demands* | |  |  |  |  |  |  |  |  |
| Low | 22.52  (21.35, 23.33) | 10.32  (9.66, 10.93) | 12.20  (11.09, 13.00) | 23.37  (22.42, 23.99) | 11.71  (11.06, 12.28) | 11.66  (10.98, 12.26) | 24.29  (22.87, 25.34) | 13.17  (12.16, 13.92) | 11.12  (9.95, 12.02) |
| High | 22.32  (18.00, 23.96) | 10.29  (8.69, 11.32) | 12.03  (9.18, 13.48) | 23.22  (21.36, 24.45) | 11.69  (10.50, 12.56) | 11.53  (10.30, 12.65) | 24.19  (22.79, 25.50) | 13.17  (12.06, 14.19) | 11.03  (9.75, 12.19) |
| *Task requirements* | |  |  |  |  |  |  |  |  |
| Low | 22.45  (21.09, 23.43) | 10.32  (9.72, 10.94) | 12.12  (11.05, 12.89) | 23.32  (22.35, 23.85) | 11.61  (11.00, 12.07) | 11.71  (11.04, 12.14) | 24.23  (22.57, 25.09) | 12.94  (11.97, 13.68) | 11.29  (10.20, 12.25) |
| High | 22.48  (17.67, 24.41) | 11.03  (8.69, 12.39) | 11.45  (8.69, 13.00) | 23.49  (21.21, 25.01) | 12.39  (11.22, 13.55) | 11.10  (9.21, 12.42) | 24.55  (22.59, 25.50) | 13.81  (12.71, 14.56) | 10.74  (9.47, 11.49) |
|  |  |  |  |  |  |  |  |  |  |
|  |  |  |  |  |  |  |  |  |  |
| *Time pressure* | |  |  |  |  |  |  |  |  |
| Low | 22.83  (21.58, 23.55) | 10.41  (9.71, 10.97) | 12.42  (11.51, 13.09) | 23.46  (22.61, 24.16) | 11.75  (11.21, 12.32) | 11.71  (10.90, 12.43) | 24.14  (23.08, 25.07) | 13.13  (12.29, 13.93) | 11.00  (10.15, 11.98) |
| High | 22.85  (18.57, 24.72) | 10.93  (9.07, 12.50) | 11.92  (9.56, 13.53) | 23.42  (20.83, 24.64) | 12.24  (10.81, 13.12) | 11.18  (9.71, 12.38) | 24.04  (22.45, 25.28) | 13.58  (12.45, 14.43) | 10.46  (9.34, 11.48) |
| **Psychosocial work resources** | | | | | | |  |  |  |
| *Variation in activities* | |  |  |  |  |  |  |  |  |
| Low | 22.47  (21.29, 23.15) | 10.22  (9.37, 10.80) | 12.25  (11.24, 12.92) | 23.28  (22.75, 23.93) | 11.60  (11.05, 12.25) | 11.69  (11.10, 12.20) | 24.17  (23.10, 25.00) | 13.05  (12.24, 13.93) | 11.12  (10.07, 11.98) |
| High | 23.34  (20.54, 25.30) | 11.19  (9.91, 12.47) | 12.15  (9.67, 13.89) | 24.19  (22.53, 25.47) | 12.62  (11.30, 13.44) | 11.58  (10.43, 13.12) | 25.11  (23.38, 26.06) | 14.11  (12.94, 15.04) | 11.00  (9.88, 11.89) |
| *Autonomy* | |  |  |  |  |  |  |  |  |
| Low | 22.94  (21.54, 23.89) | 10.00  (9.43, 10.61) | 12.94  (11.77, 14.00) | 23.88  (22.88, 24.56) | 11.28  (10.64, 11.81) | 12.60  (11.88, 13.36) | 24.84  (23.45, 25.91) | 12.60  (11.75, 13.68) | 12.23  (11.20, 12.99) |
| High | 21.91  (19.70, 22.82) | 11.03  (9.69, 11.92) | 10.88  (9.50, 11.92) | 22.85  (21.48, 23.63) | 12.31  (11.44, 13.18) | 10.54  (9.59, 11.17) | 23.80  (22.31, 24.75) | 13.63  (12.68, 14.37) | 10.16  (9.09, 11.08) |
