# Supplementary material for: Occupational Exposures Associated with Life Expectancy without and with Disability
Source: Int J Environ Res Public Health. 2020 Sep 1;17(17):6377. doi: 10.3390/ijerph17176377 (PMC7503628; doi:10.3390/ijerph17176377)
Supplement: Supplementary file 1 [file ijerph-17-06377-s001.zip › ijerph-887538-supplementary/ijerph-887538-supplementary.pdf]

International Journal of
Environmental Research and Public Health

MDPI

Supplementary Material:

Table S1. Total life expectancy, and life expectancy without and with disability in association with occupational exposure of the workers at the age of 55 years for low, intermediate and high educational level [95% CI=95% confidence interval].

	**Low educational level (n=761)**			**Intermediate educational level (n=392)**			**High educational level (n=210)**		
	**Total life expectancy in years (95% CI)**	**Life expectancy without disability in years (95% CI)**	**Life expectancy with disability in years (95% CI)**	**Total life expectancy in years (95% CI)**	**Life expectancy without disability in years (95% CI)**	**Life expectancy with disability in years (95% CI)**	**Total life expectancy in years (95% CI)**	**Life expectancy without disability in years (95% CI)**	**Life expectancy with disability in years (95% CI)**
Overall	22.40 (21.18, 23.31)	10.27 (9.66, 10.93)	12.13 (11.01, 12.86)	23.43 (22.69, 23.96)	11.79 (11.22, 12.22)	11.64 (11.11, 12.19)	24.54 (23.42, 25.32)	13.40 (12.75, 13.98)	11.14 (10.18, 11.81)
			**Physical work demands**						
Repetitive movements									
Low	22.85 (20.29, 24.66)	10.49 (9.55, 11.70)	12.37 (10.51, 13.75)	23.79 (21,79, 24.98)	11.96 (10.94, 12.82)	11.83 (10.42, 12.81)	24.81 (23.11, 26.18)	13.51 (12.54, 14.43)	11.29 (10.15, 12.31)
High	22.45 (21.28, 23.32)	10.31 (9.55, 11.09)	12.13 (11.07, 12.88)	23.36 (22.52, 23.95)	11.76 (11.07, 12.29)	11.59 (10.97, 12.17)	24.34 (23.27, 25.26)	13.30 (12.45, 14.05)	11.04 (10.04, 12.04)
Use force									
Low	23.08 (20.57, 24.36)	10.77 (9.81, 11.43)	12.31 (10.24, 13.82)	23.72 (22.48, 24.45)	12.08 (11.53, 12.70)	11.63 (10.73, 12.29)	24.37 (22.96, 25.40)	13.42 (12.55, 14.11)	10.94 (9.84, 11.82)
High	22.76 (21.03, 23.25)	10.31 (9.37, 10.86)	12.45 (11.14, 13.07)	23.56 (21.98, 24.16)	11.68 (10.91, 12.25)	11.88 (10.65, 12.45)	24.38 (22.63, 25.51)	13.09 (12.10, 13.80)	11.29 (9.79, 12.21)
Uncomfortable position									
Low	23.21 (20.93, 24.51)	10.64 (9.48, 11.54)	12.57 (11.03, 13.42)	23.84 (22.86, 24.74)	12.11 (11.46, 12.71)	11.74 (11.00, 12.68)	24.46 (23.27, 25.38)	13.59 (12.66, 14.29)	10.87 (10.05, 11.62)
High	22.57 (20.33, 23.31)	10.20 (9.37, 10.84)	12.36 (10.81, 13.13)	23.29 (22.14, 23.97)	11.68 (11.05, 12.39)	11.61 (10.64, 12.13)	24.03 (22.39, 24.94)	13.20 (12.03, 14.24)	10.82 (9.48, 11.52)
			**Psychosocial work demands**						
Cognitive demands									
Low	22.52 (21.35, 23.33)	10.32 (9.66, 10.93)	12.20 (11.09, 13.00)	23.37 (22.42, 23.99)	11.71 (11.06, 12.28)	11.66 (10.98, 12.26)	24.29 (22.87, 25.34)	13.17 (12.16, 13.92)	11.12 (9.95, 12.02)
High	22.32 (18.00, 23.96)	10.29 (8.69, 11.32)	12.03 (9.18, 13.48)	23.22 (21.36, 24.45)	11.69 (10.50, 12.56)	11.53 (10.30, 12.65)	24.19 (22.79, 25.50)	13.17 (12.06, 14.19)	11.03 (9.75, 12.19)
Task requirements									

	Low educational level (n=761)			Intermediate educational level (n=392)			High educational level (n=210)		
	**Total life expectancy in years (95% CI)**	**Life expectancy without disability in years (95% CI)**	**Life expectancy with disability in years (95% CI)**	**Total life expectancy in years (95% CI)**	**Life expectancy without disability in years (95% CI)**	**Life expectancy with disability in years (95% CI)**	**Total life expectancy in years (95% CI)**	**Life expectancy without disability in years (95% CI)**	**Life expectancy with disability in years (95% CI)**
Low	22.45 (21.09, 23.43)	10.32 (9.72, 10.94)	12.12 (11.05, 12.89)	23.32 (22.35, 23.85)	11.61 (11.00, 12.07)	11.71 (11.04, 12.14)	24.23 (22.57, 25.09)	12.94 (11.97, 13.68)	11.29 (10.20, 12.25)
High	22.48 (17.67, 24.41)	11.03 (8.69, 12.39)	11.45 (8.69, 13.00)	23.49 (21.21, 25.01)	12.39 (11.22, 13.55)	11.10 (9.21, 12.42)	24.55 (22.59, 25.50)	13.81 (12.71, 14.56)	10.74 (9.47, 11.49)
Time pressure									
Low	22.83 (21.58, 23.55)	10.41 (9.71, 10.97)	12.42 (11.51, 13.09)	23.46 (22.61, 24.16)	11.75 (11.21, 12.32)	11.71 (10.90, 12.43)	24.14 (23.08, 25.07)	13.13 (12.29, 13.93)	11.00 (10.15, 11.98)
High	22.85 (18.57, 24.72)	10.93 (9.07, 12.50)	11.92 (9.56, 13.53)	23.42 (20.83, 24.64)	12.24 (10.81, 13.12)	11.18 (9.71, 12.38)	24.04 (22.45, 25.28)	13.58 (12.45, 14.43)	10.46 (9.34, 11.48)
**Psychosocial work resources**									
Variation in activities									
Low	22.47 (21.29, 23.15)	10.22 (9.37, 10.80)	12.25 (11.24, 12.92)	23.28 (22.75, 23.93)	11.60 (11.05, 12.25)	11.69 (11.10, 12.20)	24.17 (23.10, 25.00)	13.05 (12.24, 13.93)	11.12 (10.07, 11.98)
High	23.34 (20.54, 25.30)	11.19 (9.91, 12.47)	12.15 (9.67, 13.89)	24.19 (22.53, 25.47)	12.62 (11.30, 13.44)	11.58 (10.43, 13.12)	25.11 (23.38, 26.06)	14.11 (12.94, 15.04)	11.00 (9.88, 11.89)
Autonomy									
Low	22.94 (21.54, 23.89)	10.00 (9.43, 10.61)	12.94 (11.77, 14.00)	23.88 (22.88, 24.56)	11.28 (10.64, 11.81)	12.60 (11.88, 13.36)	24.84 (23.45, 25.91)	12.60 (11.75, 13.68)	12.23 (11.20, 12.99)
High	21.91 (19.70, 22.82)	11.03 (9.69, 11.92)	10.88 (9.50, 11.92)	22.85 (21.48, 23.63)	12.31 (11.44, 13.18)	10.54 (9.59, 11.17)	23.80 (22.31, 24.75)	13.63 (12.68, 14.37)	10.16 (9.09, 11.08)
